# Advancing One Health through veterinary education: a mixed methods needs assessment for implementing a WOAH-harmonized national veterinary medicine curriculum in Ethiopia

**DOI:** 10.3389/fvets.2024.1357855

**Published:** 2024-03-18

**Authors:** Andrea L. Bessler, Armando E. Hoet, Shimelis Nigatu, Samantha Swisher, Tsegaw Fentie, Bemrew Admassu, Adugna Molla, Manon Brown, Amanda M. Berrian

**Affiliations:** ^1^Department of Veterinary Preventive Medicine, College of Veterinary Medicine, The Ohio State University, Columbus, OH, United States; ^2^College of Public Health, The Ohio State University, Columbus, OH, United States; ^3^College of Veterinary Medicine and Animal Science, University of Gondar, Gondar, Ethiopia

**Keywords:** capacity building, needs assessment, veterinary services, education, One Health

## Abstract

**Introduction:**

International organizations now actively promote and implement One Health collaborative approaches to prevent, detect, and control diseases in humans and animals, recognizing the critical importance of the veterinary and agricultural sectors. Moreover, Veterinary Services are chronically under-resourced, especially in low- and middle-income countries. Given the importance of National Veterinary Services to food security, nutrition, poverty alleviation, and global health security, strengthening veterinary capacity is a priority for the international community. The World Organisation for Animal Health (WOAH) outlines a set of minimum competencies veterinarians need to support National Veterinary Services effectively. To improve the quality of veterinary education, Ethiopia has developed a new 2020 national curriculum that is harmonized with the WOAH competencies.

**Methods:**

A mixed methods needs assessment was conducted to identify barriers and challenges that Ethiopian veterinary medicine programs have faced in implementing the new WOAH-harmonized national curriculum. Representatives from active veterinary programs granting a Doctor of Veterinary Medicine (DVM) degree were invited to share their experiences via an online survey and follow-up focus group discussion.

**Results:**

Fourteen veterinary programs, representing 93% of eligible programs nationwide, participated in the needs assessment. Quantitative analysis indicated that the most difficult topics associated with the new curriculum included Organization of Veterinary Services (Competency 3.1), Inspection and Certification Procedures (3.2), and practical applications of the regulatory framework for disease prevention and control (multiple competencies). Challenges associated with specific instructional methodologies, particularly the facilitation of off-site (private and public sector) student training, were also perceived as barriers to implementation. Focus group discussions elucidated reasons for these challenges and included limitations in faculty expertise, resource constraints (e.g., supplies, infrastructure), and access to off-site facilities for hands-on teaching.

**Conclusion:**

The results of this needs assessment will be used to identify and prioritize solutions to implementation challenges, helping Ethiopian veterinary medicine programs move the new WOAH-harmonized curriculum from theory to practice. As veterinarians are integral partners in advancing One Health, strengthening the capacity of Veterinary Services can ultimately safeguard animal and human health, grow economies, and improve lives.

## Introduction

The world’s population has grown exponentially in recent history and is projected to grow from 8 billion worldwide currently, to 9.7 billion by 2050 (1). As the world’s population continues to grow, so too does the demand on agricultural sectors, including food animal production where countries’ Veterinary Services help to support the food system, protect animal (and human) health, and promote countries’ agricultural economies. For example, Ethiopia’s population is expected to reach 205 million by 2025 from its current size at approximately 120 million (1). As a consequence, existing pressures on the agricultural system such as land scarcity, insufficient agricultural technologies, and inaccessibility of pastoral livestock farmers to animal health and veterinary services are exacerbated, making it more difficult to feed the growing population (2, 3). Furthermore, there are many threats to human and animal health around the world that encourage the emergence of infectious diseases and impact food security, including climate change, habitat modifications, broadening vector ranges, and human behaviors, among others (4–8). Low- and middle-income countries (LMIC), particularly in Africa, bear a higher disease burden and experience greater consequences of climate change (9, 10). By way of illustration, Ethiopia continues to experience more frequent and severe droughts, erratic rainfall in a country reliant upon predominantly rain-fed agriculture and persistently higher temperatures leading to heat-stress on humans and animals. These climate-related changes lead to secondary consequences such as increased occurrence and rages of vector-borne (e.g., Dengue, leishmaniasis, malaria), water-borne (e.g., rotavirus), and zoonotic (e.g., leptospirosis, Q fever, trypanosomiasis) diseases (10, 11). Thus, more strain is placed upon Ethiopia’s and other countries’ Veterinary Services, which are already beleaguered by limited resources and shortages of skilled personnel to respond to and manage contagious diseases and oversee food systems. Ameliorating these countries’ Veterinary Services is an important step for decreasing disease burdens and increasing livestock production in developing countries.

Many initiatives and programs have been instituted to address these challenges, principally focused on veterinary curricula and related training programs through a One Health lens in Africa, including Ethiopia (12–16). A few of the organizations that are involved in leading or supporting training programs or curriculum development for veterinary service professionals are the Centers for Disease Control and Prevention (CDC), the Food and Agriculture Organization of the United Nations (FAO), the United States Agency for International Development (USAID), the World Bank, and the World Organisation for Animal Health (WOAH) (17–20).

The Veterinary Education Twinning Program, sponsored by WOAH, provides an example of this targeted work. In 2015, a partnership between The Ohio State University College of Veterinary Medicine (OSU-CVM) and the University of Gondar College of Veterinary Medicine and Animal Science (UoG-CVMAS) in Ethiopia was forged. This Twinning Program involved a curriculum assessment, as well as faculty and student development through exchanges and continuing education training (21). These activities culminated in the development and implementation of a UoG-CVMAS curriculum aligned with the *WOAH recommendations on the Competencies of graduating veterinarians (‘Day 1 graduates’) to assure National Veterinary Services of quality* and the *Veterinary Education Core Curriculum* (22, 23). The newly developed UoG-CVMAS WOAH-harmonized curriculum contained all 11 specific and 8 advanced competencies, covering topics including epidemiology, transboundary animal diseases, zoonoses (including foodborne diseases), animal welfare, and food safety, among others. This new, WOAH-harmonized curriculum, the first of its kind in Africa (21), was launched in September 2017 and was intended to strengthen Ethiopia’s Veterinary Services by graduating veterinarians ready to support their country’s services.

Ethiopia’s Ministry of Education (MoE) reviews and updates the national veterinary medicine curriculum every 10 years, creating in 2019 a *National Curriculum Task Force* to lead such process. The Deans and representatives of all Veterinary Education Establishments (VEEs) in Ethiopia and other stakeholders (including the veterinary association) integrated this task force. This working group agreed to use the UoG-CVMAS WOAH-harmonized curriculum as the benchmark for the new national curriculum, approving and deploying the new academic program for implementation by all Ethiopian VEEs in November 2020.

Introducing a new, nationwide professional curriculum is complex in any setting and was expected to be especially challenging in Ethiopia because of regional variability in access to resources and personnel capacity. These challenges were compounded by recent events, including the COVID-19 pandemic and armed conflict in some regions, that further disrupted educational systems and communications (24–26). It is critical to identify and address the barriers and challenges that Ethiopian VEEs are facing in implementing the new national curriculum to graduate veterinarians who can respond to emerging disease threats, protect the food system, and strengthen Ethiopia’s agricultural economy. Therefore, the methodology described in this study uses a mixed methods approach to understand VEEs’ experience of moving from theory to practice in implementing a new curriculum and identify specific difficult content and the barriers they face. While primarily focused on the national needs assessment process to identify such obstacles, the authors will briefly discuss the next steps. Following this study, the outcomes of the needs assessment will be presented for the prioritization of potential solutions for intervention and the development of an Action Plan to accomplish harmonization with the new curriculum nationally. This systematic process from the needs assessment to Action Plan will unite Ethiopian VEEs in the delivery of their veterinary programs and graduation of high-quality veterinarians, thus strengthening Ethiopia’s veterinary services and, ultimately, advancing One Health.

## Methods

### Study design and data collection

We conducted a mixed methods needs assessment between June and November 2022 to evaluate the experiences with the adoption of the new 2020 national veterinary curriculum and identify the specific challenges that Ethiopian VEEs are facing during its implementation. The needs assessment consisted of two data collection steps: an asynchronous online questionnaire (quantitative) using Qualtrics^XM^ survey software and synchronous focus group discussions (qualitative) following survey completion. Our study followed a sequential explanatory design, in which the qualitative component sought to explain and provide further information about the quantitative results (27).

### Study area and participation criteria

**Figure 1:**
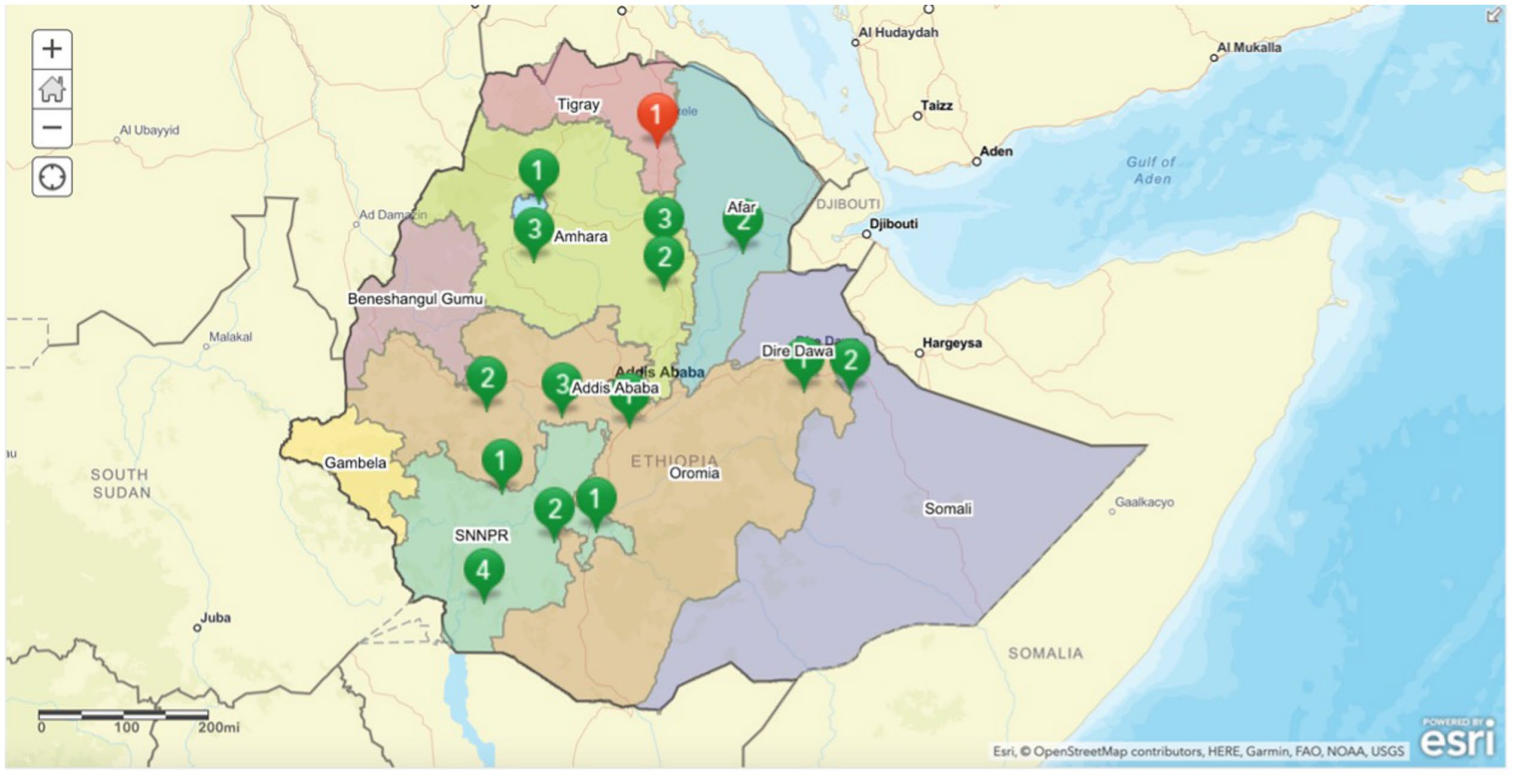
Map of participating (green) and non-participating (red) Ethiopian veterinary education establishments, including their generation number which is based on year of establishment.

Situated in the Horn of Africa, Ethiopia is an LMIC with a population of over 120 million people (second highest in Africa) (28). Agriculture is critically important to the economy of Ethiopia, comprising over 30% of its gross domestic product (29). Formal training for supporting Ethiopia’s growing agricultural sector is provided by its many universities, whose educational structure is governed by the MoE. The universities included in this study are located throughout Ethiopia, including Amhara, Afar, Oromia, Somali, and Southern Nation, Nationalities, and People’s Region (SNNPR) (see Figure 1).

The needs assessment focused on those academic programs awarding a Doctor of Veterinary Medicine (DVM) degree, as one of Ethiopia’s major personnel sources for the veterinary services workforce. All active VEEs offering a DVM degree were targeted for participation; however, very newly established VEEs (i.e., had not yet graduated a cohort of students or were unlikely to have experience with the new curriculum) were excluded from this assessment. At the time of the study, there were a total of 16 universities meeting the inclusion criteria of offering a DVM degree, 1 university was excluded due to its newly established status. Additionally, 1 eligible university was unable to participate due to conflict in their region. In total, 14 universities meeting the inclusion criteria were eligible and able to participate in the study.

### Needs assessment development

#### Online survey

The *OSU-UoG Twinning Program Action Plan* (30) was used as a starting point for the development of the needs assessment survey (step 1 of this process), as it helped to identify much of the new content that was included in the new 2020 national curriculum and most likely to create implementation issues. The UoG-CVMAS team confirmed the changes to the national curriculum and identified other topics or elements incorporated during the national curriculum task force meetings. When all new content for the 2020 national curriculum was confirmed, the identified topics and themes were transformed into questions that aimed to characterize the difficulty of implementing each identified change.

The final stage of survey development (refinement) included an external review panel, formed by VEE deans from established veterinary programs (Hawassa, Jimma, and Wollo) who were also part of the national curriculum task force. Their feedback was sought to ensure the inclusion of all topics and changes made to the new national curriculum that would need to be implemented across the VEEs and therefore need to be assessed through the survey. This feedback was used to update and create the final version that was transferred to the Qualtrics^XM^ survey platform.

The survey aimed to collect data on four main areas: (a) VEE background information, including their general experience with the implementation of the new national curriculum, (b) the level of difficulty the VEE experienced implementing the new content related to WOAH Day 1 Specific Competencies, (c) the level of difficulty implementing the new content related to WOAH Day 1 Advanced Competencies, and (d) the level of difficulty implementing cross-cutting topics/program areas (e.g., One Health, student placements for off-campus training). Excluding the background section, the survey had 36 Likert-style questions, where options for responses included: (1) does not apply to our VEE, (2) very difficult, (3) moderately difficult, (4) moderately easy (5) very easy, and (6) not sure. The selection of “does not apply to our VEE” and “not sure” produced a secondary open-ended prompt for responders to elaborate on their reason for that selection. Similarly, if responders selected “very difficult” or “moderately difficult,” a secondary prompt would encourage responders to indicate reasons for difficulty (multiple selections, including the option of ‘other’ with further specification). Selecting any response other than moderately easy or very easy would prompt further conversation during the focus group sessions.

Each participating VEE was tasked with assembling an internal “*survey team*” (discussed further below under *Needs Assessment Implementation*), which could include multiple faculty and/or administrators (Dean or Department Heads) who taught content related to WOAH Day 1 Competencies and/or participated in their 2020 curriculum implementation. Each VEE survey team was intended to both complete the online survey and participate in the focus group discussions.

#### Focus group discussions

Focus group discussions (FGDs) were designed to provide each VEE survey team the opportunity to elaborate or further explain their responses to the online survey. Individual FGDs were facilitated by members of the OSU-UoG team and conducted virtually using the Zoom platform (Version 5.13.11) with each VEE after they had completed the online survey. These confidential discussions were held in English and recorded to facilitate data collection and analysis. The FGD protocol was consistent for each VEE survey team (Table 1).

**Table 1:** Focus group discussion protocol for each Ethiopian veterinary education establishment participating in the needs assessment.

Element	Purpose
Meeting overview Goals Format	Review the objectives and purpose of the needs assessment and provide an overview and structure of the FGDs
Survey team introductions Name Role in the university (dean, lecturer, member of curriculum committee) Classes/topics taught	Each member of the survey team identified themselves, their role in the needs assessment survey, and their role within the university to better understand their perspective and experience working with the new curriculum
Background information Experience completing the survey Experience with new curriculum	Describe the survey team’s experience discussing the questions and incorporating each member’s inputs Describe the VEE’s experience with the new curriculum, including when/how it was received, guidance or instructions received, and how many years of implementation
Topics indicated “moderately difficult” or “very difficult” on survey	Review and describe topics considered difficult to implement; understand what barriers and challenges exist to warrant the difficulty
Prioritization of difficult topics	The survey team members selected and justified which identified difficult topics they would prioritize for intervention for their program
Points for clarification (e.g., does not apply, not sure)	Determine if any questions were unclear or if survey responses warranted review and further explanation
Topics indicated “moderately easy” or “very easy”	Review topics considered easy to implement; elaborate on pedagogical methods, teaching resources, other assets used/available that warranted this classification
Additional comments	Survey team members could ask additional questions or provide additional comments about implementing the new national curriculum or the needs assessment survey/process
Strategy and timeline	Provide overview of needs assessment methodology, how data will be used, and next steps to encourage transparency and continued participation

### Needs assessment implementation

Needs assessment implementation involved a series of six steps: (1) recruitment, (2) preparation, (3) online survey completion, (4) preliminary analysis, (5) focus group discussion, and (6) data integration and final analysis. The recruitment step consisted of a webinar (June 1, 2022) which served to socialize the project with the deans of recruited VEEs, who learned about the purpose of the study, the methodology, and how the results would be used to strategize and prioritize interventions. At this time, deans were tasked with assembling their internal survey teams, so the next step, preparation (step 2), could begin. Once the VEE survey teams were assembled, individual virtual meetings took place (July 2022) to describe the study methodology, its objectives and purpose, and the survey question format to each team before receiving the survey. After these pre-survey preparatory meetings, VEEs received an email with the link to the online survey, as well as a PDF version of the survey to facilitate discussion before completing and submitting the online survey (step 3), should they wish to do so. As completed surveys were received (July–November 2022), the OSU-UoG team would conduct a preliminary analysis (step 4), highlighting the elements to be addressed in the FGDs (Table 1). FGDs were then scheduled (step 5) to provide the qualitative data for the needs assessment. The sixth and final step, with the completion of all FGDs, was data integration and analysis (step 6).

### Data integration and analysis

All data were aggregated and anonymized, so no personally identifiable information was included in the analysis and presentation of results. Quantitative data management and descriptive statistics were conducted using Microsoft Excel (Version 16.73, 2023), while statistical analyses were performed using R statistical software (Version 4.2.2, 2022). FGDs were recorded and transcribed verbatim, using two auto-transcription services, Zoom (Version 5.13.11, 2023) and Mediasite (Version 8.16.0, 2023), with manual verification by the OSU-CVM team. Transcript correction, coding, and thematic analysis were performed using NVivo software (Version 20.6.2, QRS International) by multiple team members together (range: 2–4) to reduce confirmation bias. Coding was done inductively (31), utilizing the constant comparison method (32), while also listening to the meeting recordings to make any additional corrections to each transcript. Once the initial coding and thematic analysis process was complete, results were shared with the whole team (OSU-CVM and UoG-CVMAS) to discuss and validate the findings.

### Ethical considerations

The Ohio State University Office of Responsible Research Practices has determined that the referenced research does not meet the federal definition of human subjects’ research requiring review. Data was collected as part of a non-research quality improvement project, the analysis of survey data did not meet the federal definition of human subjects’ research, and currently only exists as de-identified data.

## Results

### Quantitative analysis

Fourteen (out of 15 eligible) VEEs participated in the needs assessment (both the online surveys and virtual FGDs) (93.3% response rate) (Table 2). Only one university, located in the Tigray Region, was unable to participate during the study period due to accessibility issues brought on by the armed conflict. A map indicating the participating VEEs, by generation number and region, is shown in Figure 1.

**Table 2:** Participating Ethiopian veterinary medicine programs, categorized by their year of establishment and generation.^a^

1st Generation (before 2003)	2nd Generation (2004–2011)	3rd Generation (2012–2017)	4th Generation (after 2017)
**Addis Ababa University**, College of Veterinary Medicine and Agriculture **University of Gondar**, College of Veterinary Medicine and Animal Sciences **Haramaya University**, College of Veterinary Medicine **Hawassa University**, Department of Veterinary Medicine **Jimma University**, College of Agriculture and Veterinary Medicine	**Jigjiga University**, College of Veterinary Medicine **Samara University**, College of Veterinary Medicine **Wolaita Sodo University**, School of Veterinary Medicine **Wollega University**, School of Veterinary Medicine **Wollo University**, School of Veterinary Medicine	**Ambo University**, College of Agriculture and Veterinary Science **Bahir Dar University**, School of Animal Science and Veterinary Medicine **Woldia University**, School of Veterinary Medicine	**Jinka University**, Department of Veterinary Science

a Year of establishment is based on when the university initiated its DVM granting degree program. Universities either have a separate college/school of veterinary medicine within their university or a department of veterinary medicine within their college/school of agriculture or animal science.

To quantify the survey results and identify the topics from the new 2020 curriculum that VEEs found most difficult to incorporate, a numeric value was assigned to the ordinal survey responses. For this purpose, a value of [4] was assigned to “very difficult,” “moderately difficult” [3], “moderately easy” [2], and “very easy” [1]. Topics were also organized by their corresponding WOAH Day 1 Competency (Specific and Advanced) (Table 3) or cross-cutting topic/program area (Table 4), depending on their focus.

**Table 3:** Topics and their WOAH Day 1 competency ranked from the most difficult to least difficult.

Topic	WOAH Day 1 competency	Mean difficulty (SD)
Organization of veterinary services in Ethiopia	3.1—Organization of Veterinary Services	2.67 (0.745)
Practical application of the regulatory framework for disease prevention and control	2.2—Zoonotic (and Foodborne) Diseases 2.3—Transboundary Animal Diseases 2.4—Emerging & Re-Emerging Diseases	2.29 (0.699)
Inspection and certification procedures for exportation, including international import control mechanisms for animals and animal products	3.2—Inspection and Certification Procedure 3.7—International Trade Framework	2.29 (0.699)
Proper management and use of veterinary products (i.e., milk testing for drug residues)	2.7—Veterinary Products	2.21 (0.773)
Practical applications of professional communication skills	2.11—Communication Skills	2.21 (0.674)
Practical applications of risk analysis	3.5—Applications of Risk Analysis	2.21 (0.773)
Practical applications of the understanding and applications of high standards of veterinary ethics	2.9—Veterinary Legislation and Ethics	2.14 (0.914)
Practical applications of performing physical examinations for the preparation of health certificates for animal movement	2.10—General Certification Procedures	2.14 (0.833)
Practical applications of diagnostic and therapeutic tools for disease prevention and control	2.2—Zoonotic (and Foodborne) Diseases 2.3—Transboundary Animal Diseases 2.4—Emerging & Re-Emerging Diseases	2.14 (0.742)
Practical applications of the economic and public health implications (including international trade) of diseases	2.2—Zoonotic (and Foodborne) Diseases 2.3—Transboundary Animal Diseases 2.4—Emerging & Re-Emerging Diseases	2.07 (0.703)
Pre-harvest management practices and conditions	2.6—Food Hygiene	2.00 (0.655)
Topics related to the international trade framework (i.e., import control and implications of disease on international trade)	3.7—International Trade Framework	2.00 (0.961)
Practical applications of the regulations/standards for animal production, transport, and humane slaughter	2.8—Animal Welfare	1.93 (0.799)
Food safety topics related to drug residues	2.7—Veterinary Products	1.92 (0.917)
Practical applications of outbreak investigation and disease control	2.1—Epidemiology	1.86 (0.833)
Practical applications of disease recognition	2.2—Zoonotic (and Foodborne) Diseases 2.3—Transboundary Animal Diseases 2.4—Emerging & Re-Emerging Diseases	1.86 (0.833)
Performing animal welfare evaluations and outings	2.8—Animal Welfare	1.79 (0.860)
National veterinary legislation	2.9—Veterinary Legislation and Ethics	1.79 (0.773)
Appropriate and rational use of antimicrobial drugs regarding withdrawal times and drug residues	2.7—Veterinary Products	1.71 (0.699)
Mechanisms that lead to the development of antimicrobial resistance	2.7—Veterinary Products	1.64 (0.718)
Practical applications of the management of contagious diseases (including foodborne)	3.3—Management of Contagious Diseases 3.4—Advanced Food Hygiene	1.64 (0.610)
Topics related to the international trade framework (i.e., sanitary and phytosanitary procedures, WOAH, Codex Alimentarius)	3.7—International Trade Framework	1.62 (0.487)
Post-harvest good sanitary and management practices	2.6—Food Hygiene	1.57 (0.495)
Proper management of veterinary products (i.e., drug withdrawal times)	2.7—Veterinary Products	1.50 (0.627)
Veterinary legislation rules and regulations governing the veterinary profession in Ethiopia	2.9—Veterinary Legislation and Ethics	1.50 (0.627)
Hazard Analysis and Critical Control Point (HACCP)	2.6—Food Hygiene	1.43 (0.623)
Appropriate food hygiene, food storage, and food preparation	3.4—Advanced Food Hygiene	1.29 (0.589)
Theoretical concepts of risk analysis	3.5—Applications of Risk Analysis	1.29 (0.589)
Disease control applications of epidemiology concepts	2.1—Epidemiology	1.23 (0.421)
Harvest: antemortem examination, postmortem examination, and humane slaughter	2.6—Food Hygiene	1.21 (0.558)
Practical applications of research methodology	3.6—Research	1.21 (0.410)
Relocating the Animal Welfare course to be given earlier in the curriculum	2.8—Animal Welfare	1.14 (0.515)

Topics ranked by perceived difficulty by Ethiopian veterinary medicine programs (*n* = 14) on the online survey. Response categories ranged from “very difficult” = 4 to “very easy” = 1.

**Table 4:** Curriculum elements and their associated cross-cutting topic/program area.

Curriculum elements	Cross-cutting topic/program area	Mean difficulty (SD)
Student placement—private sector agencies	External training/student rotation	2.77 (0.799)
Biological waste management	One Health	2.38 (0.738)
Student placement—public sector agencies	External training/student rotation	2.31 (0.606)
Practical applications of One Health at the human-domestic-wild animal interface	One Health	2.21 (0.558)
Environmental health	One Health	2.21 (0.773)
Concepts and applications of a One Health Approach	One Health	1.79 (0.674)
One Health for emerging/re-emerging zoonotic disease prevention and control	One Heath	1.71 (0.452)
One Health for food safety and food security	One Health	1.71 (0.589)

Curriculum elements ranked most difficult to least difficult to implement, as perceived by different Ethiopian veterinary medicine programs (*n* = 14). Response categories ranged from “very difficult” = 4, to “very easy” = 1.

**Figure 2:**
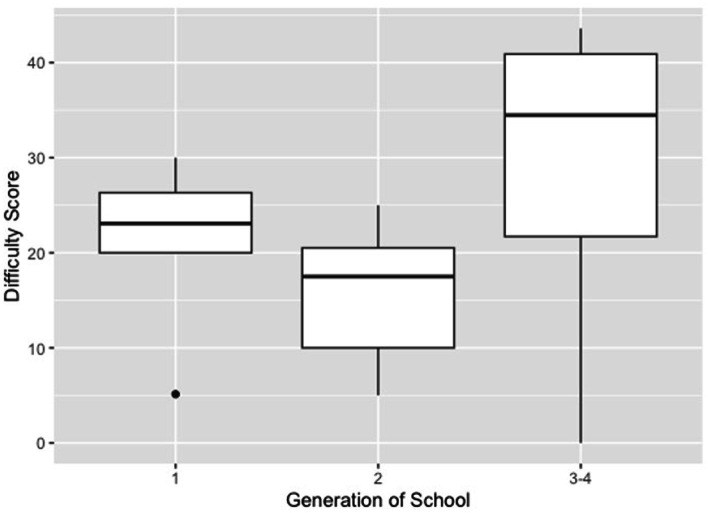
Box plots demonstrating the distribution of difficulty for incorporating certain topics into the new curriculum. Degree of difficulty is calculated as the % of questions indicated ‘Very Difficult’ or ‘Moderately Difficult’; higher scores correspond to a higher perceived difficulty.

We also evaluated the association between the perceived difficulty of implementing certain topics and VEE generation. A total of four generations (first-fourth) of VEEs were categorized based on the year their program was established (i.e., before 2003, 2004–2011, 2012–2017, and after 2017, respectively). The purpose of categorizing VEEs by generation was to identify if more senior institutions had less difficulty implementing the new curriculum than newer ones (Table 2). To do this, third- and fourth-generation veterinary academic programs were consolidated into one category and compared to first-generation and second-generation VEEs. An overall difficulty score was assigned to each VEE in which the percentage of survey responses indicated as “very difficult” and “moderately difficult” were calculated across all survey responses. Mean difficulty scores by VEE generation are presented in Table 5. Third- and fourth-generation VEEs had the highest mean difficulty rating, albeit not statistically different as compared to first- and second-generation VEEs detected/observed (Kruskal-Wallis, *p = 0.295*). There was also a wider distribution in difficulty scores for third- and fourth-generation veterinary programs compared to the first- and second-generation veterinary programs (Figure 2).

**Table 5:** Summary statistics for the degree of difficulty incorporating new topics based on the VEE generation.

Range of Individual VEE difficulty scores	Overall difficulty score	First-generation VEEs (*n* = 5) difficulty score	Second-generation VEEs (*n* = 5) difficulty score	Third- and fourth-generation VEEs (*n* = 4) difficulty score
	Mean (SD)	Mean (SD)	Mean (SD)	Mean (SD)
40	21.2 (12.4)	20.9 (8.56)	15.6 (7.21)	28.1 (17.1)

The degree of difficulty is calculated as (% of questions indicated “very difficult” or “moderately difficult”); higher scores correspond to higher perceived difficulty.

### Qualitative analysis

Major themes were organized into three categories: challenges, neutral/mixed, and strengths. Common challenges with incorporating new content were organized under seven thematic areas: (a) barriers to practical (hands-on) training (12 VEEs mentioned), (b) facilities and infrastructure limitations (11 VEEs), (c) teaching materials shortage (11 VEEs), (d) national-level challenges (11 VEEs), (e) internal and external partnership challenges (11 VEEs), (f) faculty expertise limitations (10 VEEs), and (g) demanding logistics for off-site students training (10 VEEs). Two neutral/mixed themes emerged: curriculum alignment (12 VEEs) and geographical location of VEEs (8 VEEs). Program strengths were also highlighted in the FGDs, including partnerships (13 VEEs), faculty expertise in specific content areas (8 VEEs), and other miscellaneous strengths (e.g., existing practical experiences, access to equipment, and access to facilities) (5 VEEs).

Integrating quantitative and qualitative data allowed us to generate a summary of topics that pose the greatest implementation challenges to Ethiopian veterinary medicine programs, along with their respective WOAH Day 1 Competency (or cross-cutting/programmatic) areas. Additionally, specific barriers common to 70–85% of VEEs in the country were able to be elucidated in this analysis (Table 6). Securing external (off-site) training opportunities for students, either in the public or private sector, was a commonly perceived challenge. Some specific barriers mentioned included concerns about biosecurity, disruption of operations for the hosting institution/organization, and the security of the business practices of production facilities, particularly those in the private sector. Logistics and costs of moving students to off-campus locations were also mentioned. Another common challenge was the implementation of practical (hands-on) applications of course content. Specific competencies mentioned for this challenge were related to veterinary regulations for disease prevention and control (2.2, 2.3, 2.4), professional communications (2.11), risk analysis (3.5), veterinary ethics (2.9), preparation of health certificates (2.10), and diagnostic/therapeutic tools for disease prevention and control (2.2, 2.3, 2.4). The ten topics and themes with the greatest perceived difficulty are summarized in Table 6, along with illustrative examples from FGDs.

**Table 6:** Integrated results from quantitative and qualitative data listing the ten most difficult topics to incorporate.

Topic	WOAH Day 1 competency or cross-cutting topic/program area	Mean difficulty (SD)—quantitative data	Theme(s) -qualitative data (*n* = the total number of Ethiopian veterinary medicine programs that commented)	Example from focus group discussions (VEE indicated by the number in which their FGD occurred chronologically)
Student placement—private sector agencies	External training/student rotation	2.77 (0.799)	Partnership challenges (*n* = 11) Moving students for off-site training (*n* = 10)	“[Partnership] in the private sector…an example of the dairy farms, they do not want their animals to be disturbed. They do not want the farm to be disturbed for biosecurity reasons. They do not want to disclose also their operations, how the business works and all these things. So because of this reason, we are having difficulty [placing] students in the private sector.” [FGD3]
Student placement—public sector agencies	External training/student rotation	2.31 (0.606)	Partnership challenges (*n* = 11) Moving students for off-site training (*n* = 10)	“Every year we communicate [with] different organizations, the main organizations that accept our students is the Ministry of Agriculture, where the Veterinary Services is, and especially its regional Veterinary service institutions. We communicate with the research institutes, and also regional laboratories. We write letter to the different labs where we get some response [but]. sometimes when we do not get that response, then we have to change the place” [FGD14]
Practical application of the regulatory framework for disease prevention and control	2.2—Zoonotic (and Foodborne) Diseases 2.3—Transboundary Animal Diseases 2.4—Emerging & Re-Emerging Diseases	2.29 (0.699)	Barriers to practical (hands-on) training (*n* = 12) National level challenges (*n* = 11) Faculty expertise limitations (*n* = 10)	“From the faculty’s perspective, we lack still a practical skill [of] how to do [conduct] diagnostic model for [teaching]. I think this is also a major gap in most of the faculties [veterinary schools].” [FGD1]
Inspection and certification procedures for exportation, including international import control mechanisms for animals and animal products	3.2—Inspection and Certification Procedure 3.7—International Trade Framework	2.29 (0.699)	National level challenges (*n* = 11) Faculty expertise limitations (*n* = 10) Curriculum alignment (*n* = 12)	“[…]our countries are all importing to the Middle East. [We] never export trade animal products, to Europe and Western countries because we do not have policy [for] disease eradication. We have a number of endemic animal diseases. So we are having a problem with the ability to export. And the Ministry of Agriculture, I think they are focusing to provide these certificates. And with this competency is very important for our students so that they will be skilled so they can help our nation in this certification of health, the herd level or the slaughterhouse, or our live animal export. That’s very important but challenging one as we do not have this policy of implementation with drug residue, biological management [only] is theory, but it should be a combined with a practical one.” [FGD10]
Proper management and use of veterinary products (i.e., milk testing for drug residues)	2.7—Veterinary Products	2.21 (0.773)	Facilities & infrastructure limitations (*n* = 11) Materials shortage (*n* = 11) Faculty expertise limitations (*n* = 10) Moving students for off-site training (*n* = 10)	“We did not have the facility to test veterinary product […] testing veterinary product for residues may be difficult.” [FGD8]
Practical applications of professional communication skills	2.11—Communication Skills	2.21 (0.674)	Barriers to practical (hands-on) training (*n* = 12) Faculty expertise limitations (*n* = 10)	“Actually, on the communication skill, most of the time when we say communication skill, our focus is on language.” [FGD5]
Practical applications of risk analysis	3.5—Applications of Risk Analysis	2.21 (0.773)	Barriers to practical (hands-on) training (*n* = 12) Faculty expertise limitations (*n* = 10)	“We teach the basic risk analysis methods, but we lack the application part. So application of risk analysis is very important and I think that faculty training is also very important in how to analyze risks, how to estimate and apply this case. It needs some sort of training for faculties” [FGD1]
Practical applications of the understanding and applications of high standards of veterinary ethics	2.9—Veterinary Legislation and Ethics	2.14 (0.914)	Barriers to practical (hands-on) training (*n* = 12) Faculty expertise limitations (*n* = 10)	“Veterinary Ethics and jurisprudence have been delivered by staffs with no specialization [in] this area, just it is a matter of experience. It is point raised by the school about who delivered this course so far, who has experience teaching in this area. But the person in charge [does not have] qualification in veterinary ethics and welfare unless delivering the course theoretically” [FGD2]
Practical applications of performing physical examinations for the preparation of health certificates for animal movement	2.10—General Certification Procedures	2.14 (0.833)	Barriers to practical (hands-on) training (*n* = 12) Curriculum alignment (*n* = 12) National level challenges (*n* = 11) Faculty expertise limitations (*n* = 10)	“Within the country as there is very little animal movement control. But for export purposes, there are either at postgraduate level or in the short-term training after graduation, people will get certification [training]. So after getting three months or six months training, they engage in this duty, especially with export animals. And the same is true for export abattoirs and also for the domestic abattoirs.” [FGD14]
Practical applications of diagnostic and therapeutic tools for disease prevention and control	2.2—Zoonotic (and Foodborne) Diseases 2.3—Transboundary Animal Diseases 2.4—Emerging & Re-Emerging Diseases	2.14 (0.742)	Barriers to practical (hands-on) training (*n* = 12) Partnership Challenges (*n* = 11) National level challenges (*n* = 11) Facilities & infrastructure limitations (*n* = 11), materials shortage (*n* = 11), faculty expertise limitations (*n* = 10), and moving students for off-site training (*n* = 10)	“I think there are crosscutting problems across the universities in the veterinary college pertaining to laboratory capacities in general, including source of diagnostic capacities and other major instruments for animals to do the clinical examinations. The other thing is it might be different location wise. Like students in our case, they are going out for the clinical specialty, the fourth and the fifth-year students, they are going to the clinic and the clinic [does not] have a microscope. The only thing that they do is simply based on outward signs and some physical clinical parameters as they do their diagnosis and decide the treatment without actually considering the drug withdrawal time, without considering any other drug resistance and related issues. So that makes the veterinary training very difficult. We do not have local capacities. The veterinary clinics and regional laboratories around the university are not very much well-equipped.” [FGD3]

## Discussion

Ethiopia is the first country in Africa to adopt a WOAH-harmonized veterinary curriculum to better train and equip the next generation of veterinarians who will directly support their National Veterinary Services to meet the growing needs of the country. Challenges exist, however, in moving from theory to practice in the implementation of this new curriculum across all VEEs in Ethiopia. This needs assessment identified several topics and “requirements” that have been difficult for VEEs to implement as part of their new 2020 national veterinary curriculum, as well as barriers hindering the incorporation of those topics. Among them, included the Organization of Veterinary Services (Competency 3.1), Inspection and Certification Procedures (Competency 3.2), and practical applications of the regulatory framework for disease prevention and control (multiple competencies). The FGDs expanded upon the perceived difficult topics and provided reasons for these challenges, including limitations in the facilitation of off-site (private and public sector) student training, faculty expertise on the subject matter as well as on preparing practical/applied content, resource constraints to support teaching and training (e.g., consumables, infrastructure), and access to off-site facilities for hands-on/practical teaching.

Furthermore, a commonly cited challenge acknowledged during the FGDs related to teaching materials shortage for training students. For example, some program representatives discussed challenges with the acquisition of personal protective equipment to conduct outbreak investigations, ante- and post-mortem examinations, and sample collection for infectious disease diagnostics. Others described challenges with shortages in medications, vaccinations, and other biologics used to teach their proper management and use. Similarly, significant deficiencies of laboratory materials for diagnostics, antimicrobial susceptibility testing, and veterinary product residue testing required to train future veterinarians are such important topics. Further inquiries during the FGDs identified various explanations for the materials shortages, primarily related to challenges associated with importing necessary materials into the country. While the process of supply procurement through the central government is often arduous and prolonged, financial constraints remain an enduring obstacle for veterinary programs to deliver practical or applied training. Other institutions and organizations in the region report similar challenges, citing a lack of access to adequate financial and material resources as a major impediment to One Health (33–35).

Beyond the procurement of necessary materials, financial constraints impart difficulties in implementing components of the new national curriculum, such as the organization and placement of students for off-site training. To appropriately respond to One Health issues including the frequent occurrences of disease outbreaks, antimicrobial resistance, food safety, and biosecurity, it will be critical for ministries and governmental bodies to provide access as well as establish financial and systemic support for One Health actions.

While primarily mentioned as challenges, internal and external partnerships were also cited as strengths for some programs. Many VEEs reported collaborating with other departments and colleges within their universities to fill gaps in subject matter expertise and gain access to facilities for diagnostics and laboratory techniques. For example, some programs partner with the College of Law to teach Ethics and Jurisprudence topics. Others indicated strong relationships with off-campus partners, such as abattoirs, which provided opportunities for hands-on learning and student placements for externships. These relationships varied greatly by location based on regional resource availability. Most programs reported facing challenges in securing partnerships, particularly for student placements to engage in external training in the public and private sectors. Some programs compensate for a lack of opportunities for student placements through partnerships with other universities with greater access to regional laboratories and services or with international non-governmental organizations. These results correlate with similar findings that indicate a growing need for capacity building through collaborations with national ministries, international agencies, public-private partnerships, academic institutions, One Health networks, and donor organizations (36–39).

Some of the survey result interpretations could have been impacted by deviations in the application of the surveys from the original study design. The surveys were intended to be completed by an internal survey team, representing and incorporating multiple perspectives and opinions from faculty and/or administrators; however, some survey submissions indicated a limited number of team members (the average survey team representation was 4 members, range: 1–8 members). This disparity of survey team compilation among participating VEEs could have introduced response bias in the interpretation of the quantitative results. It is possible that the surveys did not correctly identify all the difficult topics associated with the implementation of the new curriculum. However, the study design followed a mixed methodology to minimize the bias expected from individuals conducting the surveys alone. By facilitating the focus group discussions, the study aimed to capture multiple perspectives from representatives of the veterinary program who might not have been part of the initial survey team. Due to constraints of the COVID-19 pandemic and ongoing conflict in regions of the country, the ability to moderate the two study components (survey and FGDs) in person was impeded. Future studies employing this methodology should consider implementing in person moderation of the survey and FGDs whenever possible. This would help ensure the survey teams are constructed as intended and participation in both the survey and FGDs are implemented as intended to reduce response bias in either component. However, ultimately employing a mixed methods approach helps to cover gaps that either of the needs assessment components might have included in their individual application.

Similarly, the intent for the focus groups was established to encourage the group of representatives to talk with the study team and each other about their individual and shared experiences with implementing the various topics of the new 2020 national curriculum (40). However, this was not always possible because of scheduling conflicts and technology issues. This resulted in some FGDs having more limited participation and functioning more closely to a semi-structured interview, in which one or a limited number of representatives were able to participate and respond to each of the predetermined set of open-ended questions asked by the study team (41). This contrasted with our intended FGD study design, in which we hoped to provide a more open forum for representatives to freely discuss their opinions and perspectives. The shift of some of the discussions to a more semi-structured interview style might have prevented some participants from sharing their unique perspectives in front of a higher-ranking colleague. In the future, these limitations could be reduced and corrected by conducting FGDs in person to help ensure full survey team attendance and participation, help to better moderate discussions between members, and decrease constraints from technology interruptions.

Additionally, the survey results may be biased in the positive direction. Veterinary medicine programs often indicated on the survey that topics were “moderately easy” or “very easy” to implement, however during the FGDs, it was clarified that these topics are primarily being instructed at the theoretical level without practical application. This discrepancy in responses between surveys and FGDs highlight the importance of a mixed methodology in which responses can be as comprehensive as possible. However, a positive skew in survey responses might not have exposed important content that was more difficult to implement in reality, and implementing in-person moderation of surveys and FGDs would help to limit the discrepancy. Other recommendations for future studies to avoid this discrepancy and type of bias include making survey questions as short and clear as possible, avoiding leading questions, and keeping questions neutral. An example of this occurred when most programs indicated that while they teach epidemiological concepts (e.g., outbreak investigation), diagnostic techniques, and food safety topics, these lessons primarily occur in the classroom in the traditional didactic style (mostly lectures). However, to produce skilled and competent veterinarians, these academic programs lacked the incorporation of practical (hands-on) training for these topics and techniques. Depending on the veterinary program, this gap in training might be due to a lack of faculty expertise on the subject or a lack of facilities or materials to conduct training. These findings are similar to challenges identified in a previous study detailing the need for capacity building to address challenges with responding to infectious diseases in low-resource settings. Specifically, Gebreyes et al. recognized the need for standardized curricula within the One Health framework to increase the number of skilled and educated personnel, as well as the development or improvement of diagnostic laboratories as a key component of any disease surveillance, control, and prevention system (42).

An expected outcome of the needs assessment was that third and fourth-generation VEE would report greater difficulty (both in the number of topics and perceived difficulty), as they have less experience in program delivery and likely more junior faculty. However, quantitative results did not differ statistically between “older” and “newer” veterinary programs. The results did reveal a broader distribution of scores for third- and fourth-generation programs, indicating greater variability (and possibly less certainty) of responses. As newer programs gain experience in course delivery and administration, we may anticipate more consistency in the reported difficulty levels and topics, as was noted for the more established (first- and second-generation) programs.

Ultimately, the needs assessment is the first component of a larger project that seeks to help veterinary medicine programs across Ethiopia fully implement the new WOAH-harmonized national curriculum. In the next phase of this project, the identified difficult content and themes, along with the barriers recognized in the needs assessment will be presented at a multi-stakeholder national workshop. In this two-day event, participants will discuss and prioritize potential innovative solutions to the challenges identified in this needs assessment. These solutions might include faculty exchanges and continuing education, coordination of a VEE liaison with public and private organizations to improve student access and materials acquisition, or the formation of a VEE online community to exchange resources and teaching methodology similar to a community of practice framework (43). Workshop outcomes will be summarized in a comprehensive Action Plan for VEEs to consolidate the national curriculum. More information on the development and application of the workshop and its outcome will be described in a forthcoming manuscript. Nonetheless, the final product of an Action Plan is ultimately meant to support the national strategy for veterinary medicine training in Ethiopia. Ensuring that VEE programs provide a comprehensive curriculum in which veterinary graduates have received not only important foundational didactic content but also opportunities for the practical application of important topics they will apply in their roles upon graduation is necessary for the future of the veterinary workforce in Ethiopia. Successful incorporation of the WOAH Day 1 Competencies by all veterinary programs across Ethiopia incorporates a One Health approach that will help strengthen the country’s Veterinary Services, which ultimately improve animal and human health outcomes.

## Conclusion

This mixed methods needs assessment methodology identified some of the various challenges and barriers that Ethiopian veterinary medicine programs face in implementing their new 2020 national veterinary curriculum. The new veterinary medicine curriculum has been harmonized with the WOAH Day 1 Competencies for graduating veterinarians that are established to support the veterinary services of each country. It is important to support and enhance countries’ Veterinary Services as they respond to emerging/re-emerging and transboundary animal diseases, threats to food systems, and environmental changes that increase the prevalence of zoonotic diseases that have impacts on human and animal health. Therefore, enhanced capacity building and collaborations, along with strong governmental support will be necessary for LMICs, including Ethiopia, to adapt and respond to threats against animals, humans, and the environment.

## Data availability statement

The raw data supporting the conclusions of this article will be made available by the authors, without undue reservation.

## Author contributions

ALB: Conceptualization, Data curation, Formal analysis, Investigation, Methodology, Project administration, Software, Visualization, Writing – original draft, Writing – review & editing. AH: Conceptualization, Data curation, Formal analysis, Funding acquisition, Investigation, Methodology, Supervision, Writing – original draft, Writing – review & editing. SN: Data curation, Investigation, Methodology, Writing – review & editing. SS: Data curation, Formal analysis, Investigation, Methodology, Writing – review & editing. TF: Investigation, Methodology, Supervision, Writing – review & editing. BA: Data curation, Investigation, Methodology, Writing – review & editing. AM: Data curation, Investigation, Methodology, Writing – review & editing. MB: Data curation, Software, Writing – review & editing. AMB: Conceptualization, Data curation, Formal analysis, Funding acquisition, Investigation, Methodology, Project administration, Resources, Software, Supervision, Validation, Visualization, Writing – original draft, Writing – review & editing.
